# A framework for Li-ion battery prognosis based on hybrid Bayesian physics-informed neural networks

**DOI:** 10.1038/s41598-023-33018-0

**Published:** 2023-08-24

**Authors:** Renato G. Nascimento, Felipe A. C. Viana, Matteo Corbetta, Chetan S. Kulkarni

**Affiliations:** 1 Department of Mechanical and Aerospace Engineering, University of Central Florida, Orlando, FL 32816 USA; 2 KBR, Inc., NASA Ames Research Center, Moffett Field, CA 94035 USA

**Keywords:** Computational science, Software, Statistics, Mechanical engineering

## Abstract

Li-ion batteries are the main power source used in electric propulsion applications (e.g., electric cars, unmanned aerial vehicles, and advanced air mobility aircraft). Analytics-based monitoring and forecasting for metrics such as state of charge and state of health based on battery-specific usage data are critical to ensure high reliability levels. However, the complex electrochemistry that governs battery operation leads to computationally expensive physics-based models; which become unsuitable for prognosis and health management applications. We propose a hybrid physics-informed machine learning approach that simulates dynamical responses by directly implementing numerical integration of principle-based governing equations through recurrent neural networks. While reduced-order models describe part of the voltage discharge under constant or variable loading conditions, model-form uncertainty is captured through multi-layer perceptrons and battery-to-battery aleatory uncertainty is modeled through variational multi-layer perceptrons. In addition, we use a Bayesian approach to merge fleet-wide data in the form of priors with battery-specific discharge cycles, where the battery capacity is fully available or only partially available. We illustrate the effectiveness of our proposed framework using the NASA Prognostics Data Repository Battery dataset, which contains experimental discharge data on Li-ion batteries obtained in a controlled environment.

## Introduction

Electric and hybrid-propulsion systems are key enablers of advanced air mobility transformation, in which small and large aircraft will rely on Li-ion batteries to source part of all power needs. As a critical component of the powertrain, safe operation of these batteries will require robust prognosis and health management methods^1,2^. Current literature shows an array of methods for battery monitoring with models based on first principles^3,4^, machine learning^5–7^, and a combination of both^8–10^. However, existing modeling approaches often find roadblocks including: (a) governing equations are complex; and when available, high-fidelity simulations are computationally expensive to be executed onboard; (b) purely data-driven models do not necessarily obey the governing physics, nor do they generalize well to scenarios on which they have not been trained; and (c) collecting enough high-quality data to adequately train data-driven models for a complex system is often challenging—in fact, data available for adjusting reduced-order models or building machine learning models can be poor (plagued with noise, missing data, unbalanced observations of inputs and outputs, etc.). These challenges are commonly shared across many prognosis applications; creating a need for a robust modeling approach that is computationally efficient, while being grounded on first principles, and can account for unstructured datasets.

With this background, physics-informed neural networks^11–13^ have the potential to revolutionize prognosis and health management. This class of machine learning methods can potentially mitigate the lack of data as well as other problems such as poor interpretability of purely data-driven models while offering accuracy that is comparable to high-fidelity simulations at a fraction of the computational cost. In fact, recent developments in neural operators^14,15^ indicate that for problems in which the partial differential equations are known, the trained neural networks can be reused to make predictions even outside the boundary/initial conditions used in training. However, many complex systems cannot be described purely by partial differential equations, but rather by a set of governing equations and empirical laws that might not be fully characterized at the same time that available data are scarce. This explains the growing interest in hybrid physics-informed machine learning^16,17^ as a promising modeling framework for complex applications such as electric and hybrid propulsion systems. The hybrid framework we propose uses a different paradigm when compared to physics-driven loss functions as in^11^. It leverages existing equations of a system to build a model, and introduces small data-driven kernels strategically *within* the model. The data-driven portions of the model compensate for missing physics, model-form uncertainty, and ignorance of model parameters.

**Figure 1:**
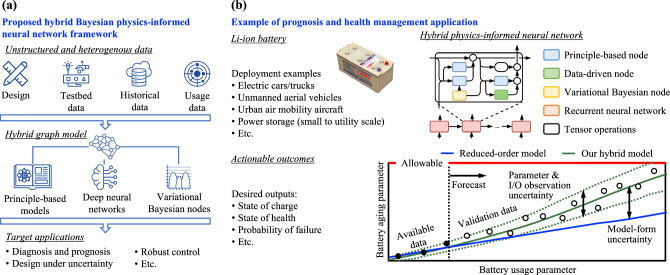
Hybrid Bayesian physics-informed neural network framework and its application to prognosis of Li-ion batteries. (**a**) Our framework directly implements numerical integration of governing equations through a hybrid graph model that merges principle-based nodes, data-driven nodes, and variational Bayesian nodes. As a result, the model can accommodate information coming from design, testbed experiments, historical and usage data in applications ranging from diagnosis and prognosis modeling, design under uncertainty, robust control, etc. (**b**) In the Li-ion battery prognosis application, the state-of-charge dynamics is modeled through a recurrent neural network such that physics-derived reduced-order models are built in as nodes; and data-driven as well as variational Bayesian nodes quantify the different forms of uncertainty in prediction. Our model can be used for discharge prediction as well as battery aging forecast far ahead in the life of the battery.

In terms of Li-ion battery prognosis, it is difficult to build models that can predict battery end of discharge while accurately accounting for the effect of battery aging. Challenges include effect of random-discharge cycles, effect of cumulative energy drawn from the battery, inter-battery variability, temperature effects during aging, and availability/completeness of recorded data. In order for prognosis models to be useful, they must furnish uncertainty estimates used in optimization of batteries operation as well as guiding model updates.

In this paper, we propose a hybrid Bayesian physics-informed neural network approach, depicted in Fig. 1a, to address the following key challenges:

- *Computational footprint*: models used for prognosis have to be computationally efficient, both in terms of memory requirement and speed, as they are often used in embedded applications or to run thousands of “what-if” scenarios. Our proposed hybrid model implements the numerical integration of governing time-dependent dynamics using recurrent neural networks^18^.
- *Partially characterized first-principle models*: in many real-world applications, degradation or failure mechanisms are only partially modeled, due to computational complexity or lack of complete knowledge. In our approach, model-form uncertainty introduced by either unknown physics or by reduced-order models is compensated with data-driven nodes placed in the design of the recurrent neural networks^19,20^.
- *Unstructured datasets*: ideally, prognosis models should be able to leverage very diverse sources of information—from legacy systems, to design, to early operation, etc. Our framework can handle heterogeneous sources of data such as design and laboratory experiments (e.g., reduced-order model and constants used therein); while also compensating for reduced number of data points with prior information coming from historical data (e.g., legacy fleet data).

The hybrid graph model described in Fig. 1a is at the foundation of the work presented here. It is composed of a series of nodes, each one corresponding to a single or multiple sets of equations. Such equations can be derived from first principles, empirical or phenomenological models, or data-driven models like deep neural networks and variational Bayesian models. Input–output relationships among the nodes are represented by edges connecting those nodes, thus blending physics-derived and data-driven blocks. Physics-derived model parameters and neural network parameters are then trained in a single stage using off-the-shelf deep learning libraries.

The incorporation of different data sources is possible, in part due to the hybrid nature of our model, which mixes principle-based and machine learning kernels. For example, the principle-based portion of the model is friendly to engineering and design information; while the machine learning portion can accommodate observed data. In addition, our Bayesian approach to data fusion makes it possible to use data that becomes available in different points during the battery life cycle.

In recent years, hybrid and physics-informed data-driven modeling strategies for prognostics have been proposed. Some^21,22^ use a mixture of physics-based models or parameters derived experimentally to feed neural networks with the intent to predict the remaining useful life of complex systems, and potentially outperform pure data-driven models. Others^23^ proposed a deep learning architecture where the last layer is tailored to solve differential equations to compute the point-solution at two time steps simultaneously, to estimate parameters of power electronic converters. An overview of methods that broadly apply to prognostics, reliability analysis as well as system safety can be found in^24^. Our prognostics framework differs from other, existing frameworks as it blends physics-derived equations and data-driven kernels in a sole hybrid model, and can train both data-driven and physical parameters together using back-propagation. Other frameworks combine physics and machine learning sequentially, where each model is calibrated/trained independently and serves as input to the other. Our framework can also accommodate physics-driven error metrics in the loss function like in^11^. In addition, model parameters can either be trained for each physical sample, e.g., train a different parameter for each battery in the training set separately, or shared across multiple samples of the same system type, e.g., train parameters to represent a fleet of similar batteries. A single model can be composed of a mix of both, parameters that are shared across different specimens, and parameters that needs to be tailored for each specimen.

Figure 1b details the application of our proposed framework to the prognosis of Li-ion batteries. The battery state of charge is approximated using discretized ordinary differential equations based on the Nerst and Butler–Volmer models^25,26^. These are at the foundation of the principle-based nodes (blue blocks), which captures the major trend of the battery state-of-charge, but discrepancies between the predictions of those models and field data prevent them from being used alone for battery risk management. Therefore, we pair the principle-based nodes in the graph with (a) purely data-driven nodes to adjust for model-form uncertainty coming from model simplifications; and (b) variational Bayesian nodes^27–29^ to account for data uncertainty coming from battery-to-battery variability and observation uncertainty. In practical terms, our hybrid model offers the following key benefits to battery operators: (1) it does not rely solely on constant discharge curves, which are the standard to estimate the battery residual capacity; and therefore, model updating can be performed without decommission of the battery; (2) it can handle battery-to-battery variation; which can happen due to factors such as inherent variability of manufacturing, initial internal damage, etc; and (3) it models battery degradation incorporating fleet-wide data; and with the aid of the Bayesian formulation, it can handle model updates with full and partial discharge cycles as well as missing history of battery usage. The approach is demonstrated using the experimental data publicly available through the NASA Prognostics Center of Excellence Data Repository^30,31^.

## Results

We present four key results: (1) estimation of model-form inadequacy in the state-of-charge model; (2) modeling of battery aging with incorporation of battery-to-battery variation; (3) model update using full and partial battery discharge cycles; and (4) ability to derive battery-specific models without requiring battery usage history.

### Handling model-form uncertainty with hybrid physics-informed neural network

The first result of the proposed hybrid physics-informed neural network is that the numerical integration of ordinary differential equations governing the state of charge is directly implemented as a recurrent neural network. This class of network models the one-step-ahead prediction of the response of interest and associated model states, given set of input values:

1 $$\begin{aligned}{}[ {\textbf{y}}_t \quad {\textbf{h}}_t ]^{\top } = f({\textbf{u}}_t, {\textbf{y}}_{t-1}, {\textbf{h}}_{t-1}), \end{aligned}$$

where the subscript *t* represents the time discretization; ${\textbf{y}} \in {\mathrm{I\!R}}^{n_{y}}$ are the observable responses; ${\textbf{h}} \in {\mathrm{I\!R}}^{n_{h}}$ are the internal states; ${\textbf{u}} \in {\mathrm{I\!R}}^{n_{u}}$ are input variables; and *f*(.) defines the transition between time steps.

**Figure 2:**
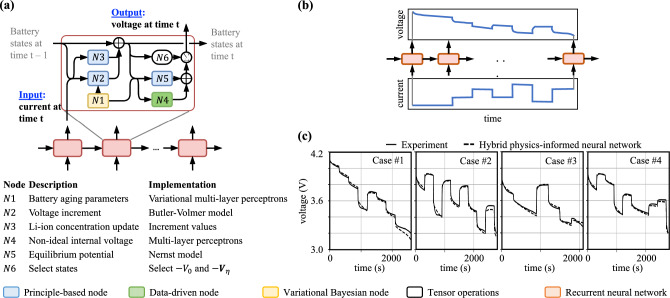
Implementing a hybrid physics-informed neural network for Li-ion prognosis. (**a**) Recurrent neural network design implements numerical integration of governing equations in the state-space representation. The recurrent unit is composed by surrogate models describing the main phenomena driving the battery electrochemistry, a data-driven node that captures the non-ideal internal voltage, and a variational Bayesian node that models aging through degradation of battery parameters. (**b**) The hybrid model takes a current time series as input and returns battery voltage. This allows one to use the model for tracking of present discharge cycles as well as forecast of future missions. (**c**) Comparison between experimental data and predictions out of our hybrid physics-informed neural network. Predictions are obtained for batteries not used in the training set.

As detailed in Fig. 2a, we propose designing *f*(.) in Eq. (1) so that (i) we implement the appropriate integration scheme^32^ (e.g., Euler, Runge-Kutta, etc.) for the set of governing equations; (ii) we use principle-based models with the intent to capture trends in the data; and (iii) we add data-driven and variational kernels to quantify model uncertainty. For the Li-ion battery model, the physics-based nodes implement the simplified electrochemistry given by the Butler–Volmer–Nernst models. The data-driven nodes estimate the non-ideal internal voltage $V_{ni, i},\,\,i=\{p. n\}$, while the variational Bayesian nodes model the total resistance $R_0$ (Ohms) and the maximum charge $q^{max}$ (Coulombs), which values are driven by the aging of the battery and may change from one specimen to another. Therefore, the data-driven and variational Bayesian nodes characterize epistemic and aleatory uncertainties in the principle-based models. The computational cost associated with our model is remarkably low. The principle-based models come from fast-to-compute reduced order models and the data-driven models are shallow Multi-Layer Perceptrons (MLPs) and variational Multi-Layer Perceptrons (vMLPs). The first-order ordinary differential equation that governs the time-dependent response is integrated using a state-space representation and is ideal for onboard computing or massive uncertainty quantification offline. Details about the implementation of the nodes are given in the Methods section and the Supplementary Material illustrate the costs associated with training and prediction.

Figure 2b illustrates the use of our hybrid models. A time series with the electric current drawn from the battery is used as input. At each time step, the hybrid physics-informed neural network updates the internal states as well as estimates the values for the voltage drop between the battery terminals. The set of hyperparameters of the hybrid model for both data-driven and variational Bayesian nodes are estimated using a set of discharge cycles. Full discharge cycles performed with fresh batteries are used in the parameter optimization of the data-driven model used to describe the non-ideal internal voltage (aging models utilize, instead, both full and partial discharge cycles). In order to ensure diversity, the training data comes from a set of batteries exposed to different duty cycles.

Finally, the trained model can then be used to predict battery discharge for batteries and missions not present in the training set. Figure 2c shows how hybrid model predictions compare against a sample of random discharge cycles. These results illustrate the model’s ability to track voltage throughout the cycle. This allows one to determine whether the battery will be able to finish a given mission; or to optimize the deployment of a set of batteries across multiple missions.

### Accounting for battery aging with variational Bayesian models

The second result of our framework is that we are able to build an aging model for a battery being monitored (“test battery”) by leveraging existing knowledge from a fleet of similar batteries. To achieve this goal, we resort to vMLPs. The model is then updated with new data coming from the test battery during operation using Bayesian update.

**Figure 3:**
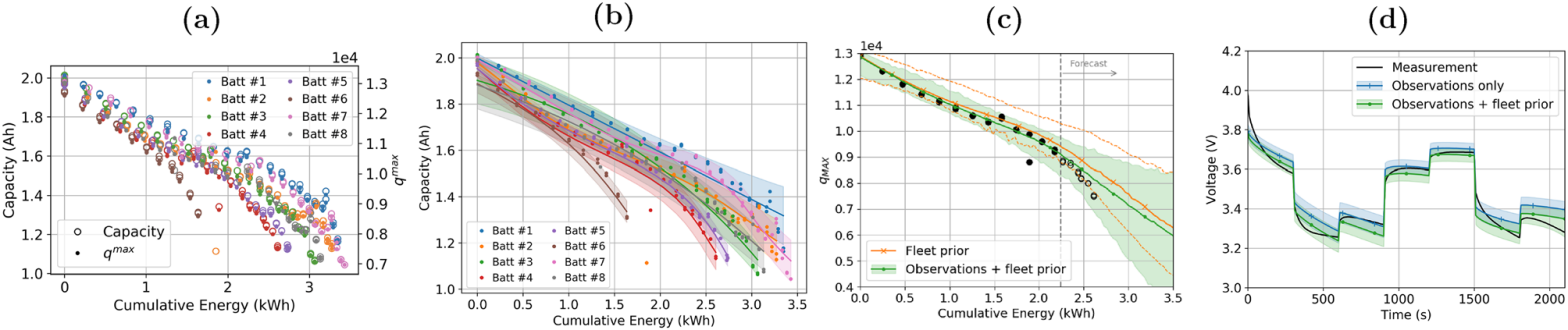
Account for battery aging using variational models. (**a**) Correlation between $q^{max}$ and *C* against cumulative energy drawn from the battery. (**b**) Individual aging models for the fleet *C*(*E*), which serve as fleet prior and are used to define the aging models $q^{max}(E)$, $R_0(E)$. (**c**) Ensemble distribution of $q^{max}$ from the fleet prior (orange lines), and posterior distribution (green line and shadowed area) computed by updating the fleet prior with estimates of $q^{max}$ visible in black circles. (**d**) The model “a-posteriori” can predict the future discharge profile at different aging stages of the battery. The prediction from the posterior model (green) is compared against predictions carried out without the fleet prior model.

First, we observed very strong correlation between capacity fading in aging batteries and changes in model parameters $q^{max}$ and $R_0$^19,26^, as shown in Fig. 3a and Fig. S4b of the Supplementary Material. We assumed that historical capacity-fading data from a fleet of older batteries can be collected and used to build aging models for each battery in that fleet. The models aim at predicting the expected value and the confidence intervals as a function of the cumulative energy drawn *E*, *C*(*E*). Each model is composed of two vMLPs to capture epistemic and aleatory uncertainty as described in the Methods section. Figure 3b shows the output of the individual variational models on top of collected capacity data. Thanks to the strong correlation observed between *C* and parameters $q^{max}$, $R_0$, we built an aging model describing $q^{max}(E)$ and $R_o(E)$, as explained in further details in the Methods section. The variational models of all other batteries in the fleet work as prior for the test battery, and they are combined in an ensemble model as follows:

2 $$\begin{aligned} q^{max}(E) = \sum _k \omega _k^{q} \, q^{max}_k(E) \qquad {\text {and}} \qquad R_0(E) = \sum _k \omega _k^{R} \, R_{0,k}(E), \end{aligned}$$

where $\omega _k^q$ and $\omega _R^q$ are the weights of *k*-th model, defined later in the Methods section; and $q^{max}_k(E)$ and $R_{0,k}(E)$ are the *k*-th vMLPs associated with batteries used to build fleet priors, as illustrated in Fig. 3b. Therefore, $q^{max}(E)$ and $R_0(E)$ represent the estimates “a priori” as an ensemble model derived from the fleet. The orange lines in Fig. 3c show an example of prior for $q^{max}$. Every time new data is collected from the operation of the test battery, either through a reference discharge cycle test or by indirectly estimating them, we update the ensemble-model prior given the predictions from the fleet, and update the weights and biases of the test battery vMLP models using Bayes’ rule. This leads to a new a-posteriori model, as depicted in Fig. 3c, green line and shaded area.

This approach allows us to leverage the existing trends of capacity fading of older batteries to infer future values of $q^{max}$, $R_0$ for the test battery. Forecast of $q^{max}$, $R_0$ at future values of *E* as the one in Fig. 3c enables the prediction of the voltage discharge behavior of the test battery at different aging stages, as shown in Fig. 3d.

### Dealing with complete and censored data through specialized loss functions and variational models

**Figure 4:**
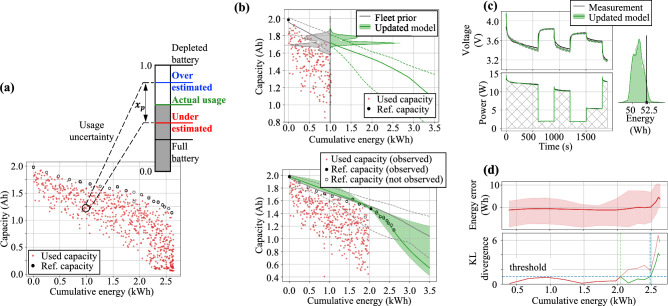
Using full and partial discharge cycles to perform model updates. (**a**) In partial discharge cycles the only available information is the used capacity; which imposes uncertainty in the estimation of available Li-ions, $x_{p,n}$. (**b**) Fleet information can be used to elucidate prior distributions; while both full and partial discharge cycles can be used for continuous model update. The top panel illustrates the update with one full discharge at $E = 0 \, {\text{kWh}}$ and partial discharge cycles between $E=0 \, {\text{kWh}}$ and $E = 1 \, {\text{kWh}}$. The bottom panel shows the update with full discharges at $E = 0 \, {\text{kWh}}$ and $E = 2 \, {\text{kWh}}$ and partial discharge cycles between $E=0 \, {\text{kWh}}$ and $E = 2 \, {\text{kWh}}$. (**c**) Uncertainty in model parameters can be propagated to state-of-charge throughout a mission (voltage history). Integration of voltage and current is used to estimate power and energy used in each cycle. The estimated energy distribution can be compared against actual used energy. (**d**) As long as the distribution of the error in the energy estimation is stationary, the hybrid model can be used for the specific battery. Metrics such as KL-divergence can be used to indicate when new full discharge cycles are needed for model updating.

The third result of the proposed hybrid physics-informed neural network is the ability to use both complete information (constant discharge) as well as censored data (random discharge) to perform battery-specific model update, as illustrated in Fig. 4a. Full discharge data are obviously more effective in reducing model-parameter uncertainties than partial discharge data. However, the fact that our framework can incorporate partial discharge implies that the models are updated while the batteries are still in use. From our experiments, we observed that model behavior is consistent with the aging effects observed in the fleet of similar batteries used for training. Thus, if the aging of a test battery follows (in a statistical distribution sense) the aging already seen in the fleet describing the training set, then partial discharge cycles are sufficient to guarantee a proper model updating and refine model predictions of future discharge cycles. However, full discharge data are likely to be necessary for test batteries behaving very differently from the one seen in the training set. The number of full discharge cycles necessary strongly depends on the behavior of the battery during operation. The update of the capacity model for a sample battery is illustrated in Fig. 4b. The available data at 1 KWh is composed of one constant discharge at 0 KWh and all random discharge cycles run up to 1 KWh. The posterior model can be used to forecast capacity as a function of cumulative energy – Updated Model in Fig. 4b. Further results on the reduction of uncertainty obtained by updating models with only partial discharge data is provided in the Supplementary Material.

In order to evaluate whether a model produces predictions accurate enough for further use, we propose estimating the distribution of the error between the predicted and consumed energy and evaluate the stationarity of such error using a statistical measure. To do so, we predict the voltage discharge curve and corresponding uncertainty coming from the model parameters. Voltage and applied (load) current profile can be integrated to estimate the energy distribution necessary for a specific mission. This energy distribution can then be compared against the actual energy consumed by the battery, as in Fig. 4c. The models can be used for prognosis for as long as the distribution of this energy error remains stationary. Metrics such as the KL-divergence between the present energy error distribution and the energy error distribution immediately after the model is updated with full discharge data can be used to indicate the need for new full discharge data, as illustrated in Fig. 4d. The top panel shows the median and the 95% confidence interval of the energy error distribution over the battery usage. While there is expected variation as the battery accumulates cycles, it is not until cumulative energy reaches 2 kWh that the energy error distribution starts to diverge substantially. This is easily captured by KL-divergence, which becomes an index for the “health of the model,” as shown in the bottom panel of Fig. 4d. The threshold level for the KL-divergence that will indicate the need for new full discharge data is application dependent and we recommend to use a fleet of batteries to determine it. In our study, we found this value to be around 1.0 (see discussion in the Supplementary Material).

### Quantifying uncertainty in aging due to unknown battery usage through Bayesian update

The fourth result of our proposed framework is the ability to perform battery-specific model update without requiring complete knowledge of past battery usage. This scenario is likely to occur when monitoring legacy fleets of battery-powered vehicles and devices. Operators, insurers, and service providers might have to serve batteries with unknown previous operations, route/usage structure and patterns, driver/user behavior, etc. Nevertheless, to control operations and maintenance costs, it is important to quantify battery degradation and estimate capacity fade over future usage. The hybrid physics-informed neural network models for such batteries can be obtained by (1) monitoring present and future decay in capacity against the observed increment in cumulative energy; and (2) performing Bayesian update of fleet-wide model taken as prior information in the light of newly recorded data.

**Figure 5:**
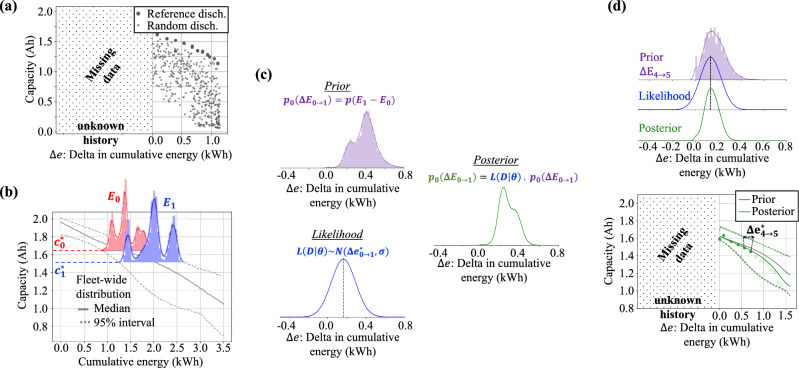
Modeling battery aging without known usage history. (**a**) Although capacity data might be missing for considerable amount of operation, once it is consistently tracked, it can be used for updating aging models. (**b**) Fleet-wide information of capacity versus cumulative energy is used to estimate cumulative energy distributions for observed capacity levels. (**c**) Bayesian update is performed on the difference between observed cumulative energy. (**d**) With enough observed capacity levels, aging models move from fleet to battery-specific distribution.

Figure 5a illustrates the case in which battery usage becomes available after a period. Without historical data, capacity decay is registered against the increment in cumulative energy from the moment the battery starts being monitored ($\Delta E$). Any two observed capacity levels and the physics-informed neural network developed with data coming from a fleet can be used to estimate the distribution of possible cumulative energy; as shown in Fig. 5b. With these two distributions, we can convert the fleet-wide model into a battery-specific one by performing Bayesian update. As depicted in Fig. 5c, we use the distributions of Fig. 5b to define the prior distribution of the increment in cumulative energy, $p_0(\Delta E_{0 \rightarrow 1})$ and the observed $\Delta E_{0 \rightarrow 1}$ to define the likelihood $L(\Delta E_{0 \rightarrow 1} | \varvec{\theta })$; where $\varvec{\theta }$ is the vector of parameters for the hybrid model. As illustrated in Fig. 5d, as more data becomes available, the fleet-wide model will converge to the battery being monitored. The battery-specific updated model is used to forecast capacity degradation; and therefore, perform optimization of battery usage throughout service life.

## Discussion

The formulation for hybrid Bayesian physics-informed neural network presented in this paper advances the field of scientific machine learning by defining a computationally efficient framework for modeling of dynamical systems (the computational footprint is illustrated in the Supplementary Material). The approach builds a directed graph model incorporating physics-based nodes that capture the trends in the data while quantifying and propagating uncertainty using data-driven nodes. These data-driven nodes are introduced into the graph in order to compensate for limitations in predictability originally found in the principle-based model as well as handling unstructured datasets, including partially observed and historical data. The framework is supported by Bayesian methods; and in the Li-ion battery prognosis application, it uses fleet-wide information to build prior distributions for battery-specific models while updating these models with operational data. Among the major advantages of the proposed framework, we can mention:

- *Quantification of model inadequacy:* our hybrid approach uses data-driven nodes to quantify model-form uncertainty. In the Li-ion battery prognosis application, the approach has showed the ability reduce the gap between predictions and observations with only a very reduced number of training points.
- *Quantification of data uncertainty:* our hybrid approach uses variational Bayesian nodes to quantify different forms of observation uncertainty. In our application, the approach successfully account for variability of aging rates of different batteries due to factors such as inherent manufacturing variability, initial internal damage, etc.
- *Robustness to unstructured data:* the Bayesian formulation allows using historical and fleet-wide data to build priors for model parameters. In practice, this reduced the need for controlled full discharge cycles normally used in precise estimation of battery residual capacity; which means that model updates can be performed without decommissioning the batteries. In addition, this prior estimates of aging behavior ensure that model update can be performed with partial discharge cycles without even requiring previous usage history of the battery.
- *Reliable tracking of model accuracy:* the approach is able to monitor the predictive capability of the model (i.e., the “health” of the model) by monitoring how stationary the integrated output errors are. In the battery application, this is done by using metrics such as KL-divergence on top of the error in energy consumption on a cycle-by-cycle basis. This can be used to inform when controlled full discharge cycles are needed for major model updates.

The proposed framework will impact onboard health monitoring systems for electric propulsion and can be directly applied to adjacent technologies, such predictive maintenance and digital twins of industrial equipment. Soft robotics is another challenging application space, in which neural networks have been used to model the complex dynamics^33^. Approaches proposed here could reduce the computational cost associated with numerical integration of the highly nonlinear material models; and therefore, enabling model-based control.

In addition, our hybrid modeling framework has the potential to substantially facilitate verification and validation of onboard analytics due to its ability to quantify and mitigate different sources of uncertainty. This capability facilitates formal verification and validation and improves compliance with norms such as DO-178^34^ and ARP-4754^35^. In turn, this helps clearing the path to certification of machine learning enabled systems in highly regulated environments, such as civil aviation.

Among the limitations of framework presented in this paper, we can mention:

- *From the modeling perspective*: our framework assumes that (a) physics-based models that should approximate the relationship between inputs and outputs of interest exist; and (b) engineers and scientists should be able to pinpoint the cause of gap between predictions and observed data. Consequently, we can implement the hybrid model and place the data-driven nodes accordingly. We assume that physics-based equations implemented in the graph will have computational cost comparable to linear algebra found in neural networks. These equations must be differentiable to enable back-propagation of gradients.
- *From the application perspective*: our approach was tested using a relatively homogeneous dataset; discharge curves came from Li-ion batteries similar to one another. In addition, all batteries used for testing were subject to similar loading conditions (i.e., either constant loading or piece-wise random loading). We anticipate that the aging behavior of batteries can change when batteries are subjected to substantially different conditions. For example, if recharging batteries that were not fully or consistently depleted can introduce further discrepancy between predictions and observations.

As potential future research, the model could be tested under realistic loading profiles coming from electric or hybrid propulsion vehicles, under varying temperature profiles representative of real operations. Our hybrid physics-informed neural network model could also be extended to other powertrain components, or even to a full hybrid powertrain model. For electric vehicle operators, a hybrid powertrain model could provide not only end-of-discharge predictions, but also fault detection and isolation within the powertrain system. Another important suggestion of future work is further quantify and improve the uncertainty prediction so that this feature of our hybrid approach can be used to accurately estimate model confidence (methods that can be used include calibration curves and metrics such as expected calibration error^36^). Finally, another venue that could be explored is the model architecture alternatives (number of layers, number of neurons, activations functions, and choice of optimizers) of the data-driven nodes. Subject to application complexity, one could pursue the optimization of the data-driven architecture alternatives with technologies such as neural architecture search^37–39^.

## Methods

### Implementing the hybrid physics-informed neural network

The hybrid physics-informed neural network proposed here implements the integration of ordinary differential equations describing battery simplified physics in the state-space form. Here we describe the simplified eletrochemistry model based on Nernst–Butler–Vomer equations^26,25,40^ as well as the data-driven and variational Bayesian nodes of Fig 2a.

#### Equilibrium potential

Based on the work presented in^25^, we approximate the equilibrium potential as:

3 $$\begin{aligned} V_{U, i} = U_0 + \frac{R\,T}{m\,F} \ln {\frac{1-x_i}{x_i}} + V_{ni,i}\qquad {\text{and}} \qquad x_i = q_i / q^{max}, \end{aligned}$$

where the subscript $i = \{ n, p\}$ indicates the negative or positive electrode, respectively; $U_0$ is the reference potential; *R* is the universal gas constant (and should not be confused with the total resistance $R_0$); *T* is the electrode temperature; *m* is the number of electrons transferred in the reaction; *F* is the Faraday constant; *x* is the mole fraction for the Li-intercalated host material; $V_{ni,i}$ is the non-ideal internal voltage and activity correction term, null in ideal conditions; *x* is the mole fraction ($x_p=0.4$ and $x_n=0.6$ for the batteries utilized in this work); and $q^{max} = q_n + q_p$ is the amount of available Li ions used to represent aging.

The total volume of the battery is split into two control volumes, bulk *b* and surface *s*, such that the diffusion rate from the bulk to the surface is:

4 $$\begin{aligned} {}&\dot{q}_{b\,s, i} = D \,(c_{b,i} - c_{s,i}), \quad c_{b,i} = \frac{q_{b,i}}{v_{b,i}}, \quad c_{s,i} = \frac{q_{s,i}}{v_{s,i}}, \quad q_p = q_{s,p} + q_{b,p}, \quad q_n = q_{s,n} + q_{b,n} \;, \\&\dot{q}_{s,p} = i_{app} + \dot{q}_{b\,s,p}, \quad \dot{q}_{b,p} = -\dot{q}_{b\,s,p}, \quad \dot{q}_{s,n} = -i_{app} + \dot{q}_{b\,s,n}, \quad {\text{and}} \quad \dot{q}_{b,n} = -\dot{q}_{b\,s,n}, \end{aligned}$$

where *D* is the diffusion coefficient and the concentration overpotential is calculated using the Nernst’s equation for the surface:

5 $$\begin{aligned} x_{s,i} = \frac{q_{s,i}}{q^{max}_{s, i}} \quad {\text{and}} \quad q^{max}_{s,i} = q^{max} \, \frac{v_{s,i}}{v_i}. \end{aligned}$$

#### Voltage increment

The surface overpotential is described by the Butler–Volmer equation^26^ such that

6 $$\begin{aligned} V = V_{U,p} - V_{U,n} - V_0 - V_{\eta ,p} - V_{\eta , n}, \quad V_{\eta ,i} = \frac{RT}{F\alpha }\,{\textrm{arcsinh}}\left( \frac{J_i}{2J_{i0}}\right) , \quad V_0 = R_0 \,\, i_{app} \end{aligned}$$

where $\alpha$ is a symmetry factor, $J_i$ is the current density and $J_{i0}$ is the exchange current density, while $R_0$ is the Ohmic lumped resistance used to represent aging.

#### Non-ideal internal voltage

The assumption of unity activity coefficients is not applicable to real batteries; as a result, in this paper, the non-ideal internal voltage is modeled through a multi-layer perceptron (MLP):

7 $$\begin{aligned} V_{ni, n} = {\textrm{MLP}}_n(x_n; \, \textbf{w}_n, \textbf{b}_n) \quad {\text {and}} \quad V_{ni, p} = {\textrm{MLP}}_p(x_p; \, \textbf{w}_p, \textbf{b}_p), \end{aligned}$$

where $\textbf{w}_n$, $\textbf{b}_n$, $\textbf{w}_p$, and $\textbf{b}_p$ are the MLP parameters; and subscripts *p* and *n* refer to positive and negative electrode, respectively. The set of equations (3)-(7) define the nodes implemented in the hybrid physics-informed neural network for battery prognostics.

### Accounting for battery aging with variational models

Battery aging is modeled through capacity decay over the useful life, and we use parameters $q^{max}$ and $R_0$ as proxies for the aging effect on the capacity. The input variable to the aging models is the cumulative energy *E* drawn from the battery. Both parameters $q^{max}$, $R_0$ are strongly correlated to *C*; and thus, we modeled *C*(*E*) through:

8 $$\begin{aligned} {}&C(E) \sim \mathcal {N}(\mu _C(E), \sigma ^2_C(E))\,, \quad q^{max}(E) = \alpha \,C(E), \quad R_0(E) = \frac{q^{max}(E)}{\gamma (E)},\\&\mu _C(E) = \textrm{vMLP}_{\mu _C}(E; \, \textbf{w}_{\mu _C}, \textbf{b}_{\mu _C}) \quad \text {and} \quad \sigma ^2_C(E) = \textrm{vMLP}_{\sigma ^2_C}(E; \, \textbf{w}_{\sigma ^2_C}, \textbf{b}_{\sigma ^2_C}) \end{aligned}$$

where $\alpha$ is a scaling factor, determined with the data shown in Fig. 3a; and $\gamma (E)$ is a linear model, reported in the Supplementary Material. The variational models for *C*(*E*), and as a consequence for the aging parameters $q^{max}$ and $R_0$, are composed of one vMLP to predict the mean value of the quantity of interest (epistemic uncertainty), and another one to estimate the confidence interval of the prediction (aleatory uncertainty).

Each weight and bias of the vMLPs are described by a normal distribution. This way, for the *i*-th layer of the $\mu _C(E)$ vMLP:

9 $$\begin{aligned} \textbf{w}_{\mu _C,i} \sim \mathcal {N} \left( \varvec{\mu }_{\textbf{w}_{\mu _C,i}}, \; \varvec{\sigma }^2_{\textbf{w}_{\mu _C,i}} \right) , \quad \textbf{b}_{\mu _C,i} \sim \mathcal {N} \left( \varvec{\mu }_{\textbf{b}_{\mu _C,i}}, \; \varvec{\sigma }^2_{\textbf{b}_{\mu _C,i}} \right) , \end{aligned}$$

and similarly, for the *i*-th layer of the $\sigma ^2_C(E)$ vMLP:

10 $$\begin{aligned} \textbf{w}_{\sigma _C,i} \sim \mathcal {N} \left( \varvec{\mu }_{\textbf{w}_{\sigma ^2_C,i}}, \; \varvec{\sigma }^2_{\textbf{w}_{\sigma ^2_C,i}} \right) , \quad \textbf{b}_{\sigma _C,i} \sim \mathcal {N} \left( \varvec{\mu }_{\textbf{b}_{\sigma ^2_C,i}}, \; \varvec{\sigma }^2_{\textbf{b}_{\sigma ^2_C,i}} \right) , \end{aligned}$$

The means and standard deviations of these distributions are optimized during training. The loss function is defined in Eq. (12).

Our observation is that aging is best modeled through battery specific $q^{max}$ and $R_0$. When building a model for a newly deployed battery, such that constant reference discharge data is not enough to build accurate vMLP models; we suggest using an ensemble of fleet-wide models to build priors for the new battery. The ensemble prior and corresponding weights follow:

11 $$\begin{aligned} C_{WA}(E) = \sum _i \omega _i C_i(E)\; ; \quad \omega _i = \frac{ \Sigma ^{-1} \varvec{1} }{ \varvec{1}^{\top } \Sigma \varvec{1} } \end{aligned}$$

where: $\Sigma _{i,j} = \varvec{\varepsilon }_i^{\top } \varvec{\varepsilon }_j$, and $\varvec{\varepsilon }_i$ is an error measure between model *i* and the observed values. In this paper, we used the negative log-likelihood $\Lambda _{NLL}$ as defined in Eq. (12) so that both observed and used capacities can be used in the model update. Alternatively, when observed capacity data is plentiful, the mean-squared error between the observed and the predicted capacities can be used.

**Figure 6:**
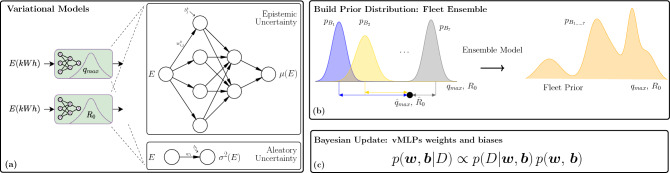
Ensemble models used to predict battery aging based on Bayesian update. The models for $q^{max}$, $R_0$ are built using variational inference-based layers, and composed of two MLPs each; one to estimate the expectation as a function of cumulative energy, and one to estimate the aleatory uncertainty, (**a**). Each battery has its own set of independent models for $q^{max}$, $R_0$. These models are combined in an ensemble model predicting the distribution $q^{max}$, $R_0$ for each level of cumulative energy draw, (**b**). Bayesian update is used to adjust the MLP parameter values as more capacity data *D* are collected, (**c**).

Figure 6 shows a graphical representation of the variational models: from vMLPs to fleet prior to Bayesian updating.

### Dealing with complete and censored data through specialized loss functions and variational models

Model update is performed using (i) fleet-wide information for definition of model-parameter priors; and (ii) partial discharge cycles, coming from step-wise random variations of input current, which only gives an estimate of used capacity. Similarly to classic variational neural networks, the loss function that drives the optimization of the vMLP hyperparameters is the sum of negative log-likelihood and the Kullback–Leibler (KL) divergence^41^:

12 $$\begin{aligned} {}&\Lambda = \Lambda _{NLL} + \Lambda _{D_{KL}}, \quad \Lambda _{NLL} = - \sum _i{\log \left( \phi _{C,i} \right) } - \sum _j{ \log \left( 1 - \Phi _{C,j} \right) }, \quad \Lambda _{D_{KL}} = \sum _i{\sum _k{\log \left( L_{i,k}\right) - \log \left( p_{0,k}\right) }}, \\&\phi _{C,i} = \phi _C\left( c_i; \; \textbf{w}_{\mu _C}, \, \textbf{b}_{\mu _C}, \, \textbf{w}_{\sigma ^2_C}, \, \textbf{b}_{\sigma ^2_C} \right) , \qquad \Phi _{C,j} = \Phi _C\left( c_j ; \; \textbf{w}_{\mu _C}, \, \textbf{b}_{\mu _C}, \, \textbf{w}_{\sigma ^2_C}, \, \textbf{b}_{\sigma ^2_C} \right) , \\&L_{i,k} = L \left( c_i, \, \textbf{w}_{\mu _C,k}, \, \textbf{b}_{\mu _C,k}, \, \textbf{w}_{\sigma ^2_C,k}, \, \textbf{b}_{\sigma ^2_C,k} \right) , \quad \text {and} \quad p_{0} = p_0 \left( \textbf{w}_{\mu _C,k}, \, \textbf{b}_{\mu _C,k}, \, \textbf{w}_{\sigma ^2_C,k}, \, \textbf{b}_{\sigma ^2_C,k} \right) \end{aligned}$$

where $\Lambda _{NLL}$ is the negative log-likelihood contribution computed using observed capacity (constant discharge) and used capacity (random discharge); $\Lambda _{D_{KL}}$ is the KL-divergence contribution computed across the vMLP layers, indicated by the subscript *k*; $\phi _C(.)$ and $\Phi _C(.)$ are the probability density and cumulative distribution functions of the normal distribution; $c_i$ and $c_j$ are the observed and used capacities, respectively; *L*(.) is the likelihood of the vMLP weights and biases; and $p_0(.)$ is the probability density function of the prior distribution of the vMLP weights and biases.

**Figure 7:**
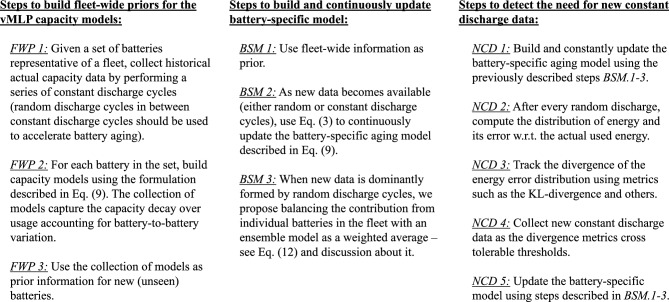
Set of algorithms used for updating battery-specific models using both full and partial discharge cycles. Left panel describes the steps for elucidation of prior distribution of the vMLP model parameters. In most practical applications, reference discharge data can be assumed to be previously available (either through battery manufacturer testing data; or by collecting historical data). Center panel details the steps for model updating. Battery-specific data is used to drive Bayesian update. However, in our study, we found that a heuristic to balance the fleet information using a weighted average ensemble^42^ might be needed when data is still poor and formed mostly by partial discharge cycles. The right panel describes the algorithm for monitoring the “health of the model.” Significant reduction in model-parameter uncertainty is expected after adding new constant discharge data. However, the cost associated with this information justify the effort in tracking model performance and only take the battery out of commission to obtain constant discharge cycles when needed.

Assuming available fleet-wide information, we build priors for the vMLP capacity models using the steps described in the left panel of Fig. 7. These fleet-based priors are used to estimate model parameters for new batteries when full discharge cycles are sparse and most data comes from regular battery usage. We assume that an initial constant discharge is performed prior to deploying the battery into service. For a new battery, not included in the set used to generate fleet priors, partial discharge cycles provide used capacity and feed the continuous update of battery-specific models. The center panel of Fig. 7 describe the algorithm we propose to perform this task. The quantified uncertainty affecting model-parameters is propagated through the hybrid physics-informed neural network and can be used guide acquisition of new constant discharge data. The proposed algorithm, detailed in the right panel of Fig. 7, calculates the need for new constant discharge cycle. Batteries submitted to constant discharge cycles are depleted completely; and therefore, the observed battery capacity has a much stronger contribution to the loss function, Eq. (12), when compared to used capacity found in regular partial discharge data.

### Quantifying uncertainty in aging due to unknown battery usage through Bayesian update

For many fielded batteries, the usage history might not be known, as depicted in Fig. 5a. Assuming that (1) there is no practical way to assess what is the battery initial capacity and cumulative energy; and (2) we can track battery capacity versus the cumulative energy differential with respect to an arbitrary point; we can write:

13 $$\begin{aligned} C(\Delta e) \sim \mathcal {N}\Big (\mu _{C}( \Delta e), \; \sigma ^2_{C}( \Delta e )\Big ), \end{aligned}$$

where *C* is the battery capacity, $\Delta e$ is the increment in cumulative energy from the moment the battery starts being monitored; and $\mu _{C}(.)$ and $\sigma _{C}(.)$ are the parameters that define the map between cumulative energy increment into battery capacity.

We propose using a Bayesian approach to update the uncertainty about battery capacity as a function of battery usage:

14 $$\begin{aligned} p(\Delta E(C; \varvec{\theta }) \; | \; \textbf{D}) = \frac{L(\textbf{D} \; | \; \varvec{\theta }) \; p_0(\Delta E)}{\int L(\textbf{D} \; | \; \varvec{\theta }) \; p_0(\Delta E) \; d\varvec{\theta }} \; ; \end{aligned}$$

where $\Delta E(.)$ is the random variable that defines the increment in cumulative energy as a function of *C* and $\varvec{\theta }$; *C* is the battery capacity; $\varvec{\theta } = [\textbf{w}_{\mu _C}, \textbf{b}_{\mu _C}, \textbf{w}_{\sigma ^2_C}, \textbf{b}_{\sigma ^2_C}]$ is the vector of parameters for vMLP models that define *C*(*E*). $\textbf{D}$ is the set of observed data; which in this case is the set of observed capacity and cumulative energy increment pairs observed since the battery started being monitored; *p*(.) and $p_0(.)$ are the posterior and prior distributions for $\Delta E(.)$; and *L*(.) is the likelihood model.

Equation (14) is known to be hard to solve due to the integral in the denominator. In order to sample the posterior distribution, here, we use numerical integration through particle filtering^43,44^. For convenience, here, we assume the likelihood to be Gaussian. Our Bayesian update strategy is illustrated in Figs. 5c, d.

## Data Availability

The data set related to this article is open source, and can be found at the NASA Prognostics Center of Excellence Data Repository, under set number 5, “Battery Data Set” and number 11, “Randomized Battery Usage Data Set”^30,31^.

## Code availability

Our implementation is all done in TensorFlow^45^ using the Python application programming interface. The code needed for replication of results is publicly available on GitHub: https://github.com/nasa/Li-ion-Battery-Prognosis-Based-on-Hybrid-Bayesian-PINN.
